# Profilaxia do tromboembolismo venoso em cirurgia bariátrica

**DOI:** 10.1590/1677-5449.041017

**Published:** 2017

**Authors:** Winston Bonetti Yoshida

**Affiliations:** 1 Universidade Estadual Paulista – UNESP, Faculdade de Medicina de Botucatu, Departamento de Cirurgia e Ortopedia, Botucatu, SP, Brasil.

Na nona edição do Consenso do American College of Chest Physicians (ACCP) de 2012, a cirurgia bariátrica foi definida como sendo de alto risco para o desenvolvimento de tromboembolismo venoso **(**TEV), juntamente com a cirurgia para o câncer ginecológico, pneumectomia, craniotomia, trauma encefálico, de medula espinal, ou outros traumas maiores^1^. Nessa categoria, a incidência de trombose venosa profunda (TVP) sem profilaxia pode variar de 40-80% distais nas pernas, 10-20% proximais, com 0,2-5,0% de incidência de embolia pulmonar (EP) fatal^2^. Dados de cirurgia bariátricas corroboram esses achados, com frequências de 0,2-2,4% de TVP e 1-2% de EP^3^^-^ 6, sendo esta última responsável por 30-50% da mortalidade operatória^7^^-^ 10. Sendo assim, a profilaxia é mandatória para prevenção do TEV.

Em metanálise de Becattini et al.^11^, os esquemas mais usados para pacientes bariátricos foram:

1. Heparina não fracionada (HNF) – na dose de 5.000 unidades internacionais (UI) subcutânea (SC) 3x ao dia por 15 dias.
2. Heparina de baixo peso molecular (HBPM) – enoxaparina na dose de 30 mg SC/2x ao dia ou 40 mg/2x ao dia por 15 dias.

O uso de doses ajustadas por Anti-Xa provocou aumento da frequência de sangramentos sem reduzir a de TEV^11^. De qualquer forma, a profilaxia farmacológica deve ser sempre individualizada, considerando o risco de sangramento de cada caso (úlcera péptica ativa, hipertensão arterial sistêmica não controlada, coagulopatia, plaquetopenia, insuficiência renal, etc.)^12^.

A enoxaparina pode ser usada na dose de 60 mg/2x ao dia por 14 dias^12^. Em comparação com a dose de 40 mg/2x ao dia por 14 dias, um estudo observou que o risco de sangramento foi similar nos dois grupos. Nesse estudo, não foi avaliada a frequência de TEV no pós-operatório.

Em junção de dados feita por Stroh et al.^13^ na Alemanha, com 31.668 cirurgias (13.772 by-passes Y de Roux, 11.840 gastrectomias em luva e 3.999 bandagens gástricas), a TVP ocorreu em 0,07% dos casos e EP em 0,10%, tendo sido preferencial o uso de HBPM. Esses dados foram corroborados por um levantamento realizado por outros autores^14^. Nesse levantamento, cerca de 94,4% das cirurgias foram abertas em Y de Roux e 5,6% por via laparoscópica.

Alguns autores preferem associar HNF com o uso de profilaxia mecânica, como deambulação precoce, compressão pneumática intermitente (CPI) e meia elástica de compressão graduada (MECG)^12^^,^ 15.

Em estudo randomizado comparando o fondaparinux (5 mg/dia) com a enoxaparina (40 mg/2x ao dia)^16^, os resultados de complicações (TEV) no pós-operatório foram similares nos dois grupos com índice de massa corporal (IMC) > 40 kg/m^2^. O fondaparinux, no entanto, teve melhor ajuste aos níveis de anti-Xa. Sendo assim, aparentemente a enoxaparina da dose de 40 mg/2x ao dia ou o fondaparinux (5mg/dia) pareceram ser recomendáveis nos pacientes com IMC > 40 kg/m^2^. Nos pacientes com IMC de 30-40 kg/m^2^ e > 60 kg/m^2^, ainda não há clara definição de dose^16^.

Em revisão recente da literatura feita por Vandiver et al.^17^, ficou clara a falta de evidências científicas quanto ao tipo e regime de doses específicos para pacientes bariátricos. As doses de 40 mg/2x dia de enoxaparina seriam efetivas e seguras para pacientes com IMC > 40 kg/m^2^ 18 ^-^ 20. De acordo com a revisão citada acima^17^, o esquema de dosagem de 0,5 mg/kg de enoxaparina teria alguns inconvenientes, pois foi bem menos validado e seria mais propenso a erros de dosagem, visto que a enoxaparina vem acondicionada em seringas prontas com doses fixas, sendo difícil seu fracionamento. Nessa revisão, a dosagem indicada de HNF foi de 7.500 UI via SC (2 ou 3x ao dia), com base em estudo retrospectivo com grande número de pacientes^20^. Ainda nessa revisão, a dose recomendada de fondaparinux foi a de 2,5 mg SC ao dia; mas, no estudo de Steele et al.^16^, a dose recomendada foi de 5 mg SC ao dia.

A profilaxia deve ser iniciada já no primeiro dia de internação, ou seja, tão logo haja risco para o desenvolvimento de TEV (ver fatores de risco). A maioria dos autores tem feito a profilaxia farmacológica somente depois da cirurgia^12^^,^ 15 ^,^ 16; porém, diante de imobilidade ou outros fatores de risco associados, esquemas de profilaxia mecânica foram indicados (CPI ou MECG).

A maior parte dos autores recomenda extensão da profilaxia por 2 semanas após a alta^17^.

Além da obesidade e da própria cirurgia bariátrica, vários fatores de risco podem estar associados e devem ser considerados com relação ao esquema de dose e de profilaxia mecânica adicional^21^:

1. História prévia de tromboembolismo
2. Trombofilia conhecida
3. Imobilidade
4. Idade > 40 anos
5. Insuficiência cardíaca congestiva
6. Doença pulmonar obstrutiva crônica
7. Uso de estrógenos
8. Insuficiência venosa crônica
9. Fraturas
10. Infecção grave
11. Doenças inflamatórias
12. Varizes
13. Câncer
14. Quimioterapia

Em vigência de profilaxia farmacológica, a anestesia raquimedular deve ser feita somente 12 horas após a última dose de enoxaparina e reiniciada somente 12 horas após a cirurgia^21^. No caso de cateter epidural, este não deve ser removido ou implantado dentro de 12 horas da última dose de HBPM e 36 horas após uso de fondaparinux, e a dose de profilaxia deve ser postergada para 2 horas após o implante ou remoção do cateter.

Ainda faltam estudos de escalonamento de doses e comparativos entre os agentes profiláticos para melhor definição do esquema profilático ideal para pacientes bariátricos.
