# Supplementary material for: Profilaxia do tromboembolismo venoso em cirurgia bariátrica
Source: J Vasc Bras. 2017 Apr-Jun;16(2):85–7. [Article in Portuguese] doi: 10.1590/1677-5449.041017 (PMC5915854; doi:10.1590/1677-5449.041017)
Supplement: Translation

# Translation: Venous thromboembolism prophylaxis in bariatric surgery

**Authors:** Winston Bonetti Yoshida

**Affiliations:** 1 Universidade Estadual Paulista – UNESP, Faculdade de Medicina de Botucatu, Departamento de Cirurgia e Ortopedia, Botucatu, SP, Brazil.

Bariatric surgery was defined as high risk for development of venous thromboembolism (VTE) in the 2012 ninth edition of the American College of Chest Physicians’ (ACCP) Consensus, along with surgery for gynecological cancer, pneumonectomy, craniotomy and traumas involving the brain or spinal cord, or other major traumas.^1^ Without prophylaxis, the incidence of deep venous thrombosis (DVT) in this risk category ranges from 40 to 80% distally in the leg and from 10 to 20% proximally in the thigh, with a 0.2-5.0% incidence of fatal pulmonary embolism (PE).^2^ Data for bariatric surgery patients corroborate these findings, with DVT frequencies of 0.2-2.4% and PE rates of 1-2%,^3^^-^ 6 the latter being responsible for 30-50% of mortality related to the operation.^7^^-^ 10 In view of this, prophylaxis for VTE is mandatory.

A meta-analysis conducted by Becattini et al.^11^ found that the regimens most often used for bariatric surgery patients were as follows:

1. Unfractionated heparin (UFH) – at a dosage of 5,000 international units (IU) subcutaneously (SC) 3 times a day for 15 days.
2. Low molecular weight heparin (LMWH) – enoxaparin at a dosage of 30 mg SC twice a day or 40 mg twice a day for 15 days.

Adjusting doses for Anti-Xa provoked an increase in the frequency of bleeding without reducing VTE.^11^ Irrespectively, pharmacological prophylaxis must always be managed on a case-by-case basis, considering the risk of bleeding in each case (active peptic ulcer, uncontrolled systemic arterial hypertension, coagulopathy, thrombocytopenia, renal failure, etc.).^12^

Enoxaparin can be administered at a dosage of 60 mg twice a day for 14 days.^12^ A study that compared this with a dosage of 40 mg twice a day for 14 days found that the risk of bleeding was similar in both groups. This study did not assess VTE frequency during the postoperative period.

Stroh et al.^13^ analyzed registry data compiled in Germany on 31,668 surgeries (13,772 Roux-en-Y-gastric bypasses, 11,840 sleeve gastrectomies and 3,999 gastric bandings) reporting DVT in 0.07% of cases and PE in 0.10% and concluding that LMWH was preferable to unfractionated heparins. These data were recently corroborated by an analysis conducted by different authors.^14^ In these data, around 94.4% of procedures were Roux-en-Y open surgeries and 5.6% were laparoscopies.

Some authors prefer to combine HNF with mechanical prophylaxis, such as early mobilization, intermittent pneumatic compression (IPC), and graduated elastic compression stockings (GECS).^12^^,^ 15

In a randomized study comparing fondaparinux (5 mg/day) with enoxaparin (40 mg twice a day),^16^ the results for complications (VTE) during the postoperative period were similar in two groups with body mass index (BMI) > 40 kg/m^2^. However, fondaparinux was associated with better control of anti-Xa levels. Apparently, therefore, either enoxaparin at a dosage of 40 mg twice a day or fondaparinux (5mg/day) can be recommended for patients with BMI > 40 kg/m^2^. There is not yet a clearly-defined dosage for patients with BMI in the range 30-40 kg/m^2^ or > 60 kg/m^2^.^16^

A recent literature review conducted by Vandiver et al.,^17^ revealed a clear lack of scientific evidence to support specific types of regimen and dosages in bariatric patients. An enoxaparin dosage of 40 mg twice a day appears to be effective and safe for patients with BMI > 40 kg/m^2^.^18^^-^ 20 According to the same review mentioned above,^17^ the 0.5 mg/kg enoxaparin regimen is associated with a greater degree of inconvenience, since it has been validated much less and is prone to dosage errors, and because the presentation of enoxaparin is in ready-to-use syringes in fixed doses, making it difficult to divide into smaller doses. The UFH dose recommended in the review was 7,500 IU SC (2 or 3 times a day), based on a retrospective study with a large number of patients.^20^ Still with relation to the same review, the recommended dose of fondaparinux was 2.5 mg SC per day; but a study published by Steele et al.^16^ recommended a dose of 5 mg SC per day.

Prophylaxis should be started on the first day of admission, i.e. as soon as there is a risk of development of VTE (see the risk factors). The majority of authors describe giving pharmacological prophylaxis only after surgery;^12^^,^ 15 ^,^ 16 but when immobility or other risk factors were also present, mechanical prophylaxis methods were prescribed (IPC or GECS).

The majority of authors recommend continuing prophylaxis for 2 weeks after discharge.^17^

In addition to obesity and the bariatric surgery itself, several other risk factors can be linked with VTE and should be taken into consideration in relation to decisions on dosage regimen and additional mechanical prophylaxis:^21^

1. Prior history of thromboembolism
2. Known thrombophilia
3. Immobility
4. Age > 40 years
5. Congestive heart failure
6. Chronic obstructive pulmonary disease
7. Use of estrogens
8. Chronic venous insufficiency
9. Fractures
10. Severe infection
11. Inflammatory diseases
12. Varicose veins
13. Cancer
14. Chemotherapy

For patients already on pharmacological prophylaxis, spinal anesthesia should only be given 12 hours after the last dose of enoxaparin, which should only be restarted 12 hours after surgery.^21^ When an epidural catheter is used, it should not be placed or removed within 12 hours of the last dose of LMWH or 36 hours after the last time that fondaparinux was administered, and prophylactic doses should be delayed until 2 hours after placement or removal of the catheter.

Dose escalation studies and studies comparing different prophylactic agents are still needed to better define the ideal prophylactic regimen in bariatric patients.
